# Linezolid and Ciprofloxacin-Induced SJS/TEN (Stevens-Johnson Syndrome/Toxic Epidermolysis Necrosis) Overlap in a Patient With Borderline Personality Disorder During a Single Hospital Stay: A Difficult Case to Manage

**DOI:** 10.7759/cureus.39242

**Published:** 2023-05-19

**Authors:** Shafia Memon, Najia Ahmed, Mohammad Nasir Memon, Fatima Zahoor, Ghazal Afzal

**Affiliations:** 1 Dermatology, Bahria University Medical and Dental College/PNS Shifa Hospital, Karachi, PAK; 2 Chief Executive, Get Solutions 360, Karachi, PAK

**Keywords:** ciprofloxacin, linezolid, bipolar personality disorder, scorten, adverse drug reaction, sjs/ten overlap, toxic epidermolysis necrosis, steven-johnson syndrome

## Abstract

SJS/TEN (Stevens-Johnson syndrome/toxic epidermolysis necrosis) is a T-cell mediated hypersensitivity syndrome in which cytotoxic CD8+ cells react against keratinocytes, resulting in widespread apoptosis and cell necrosis. About 90% of these cases are attributed to drug reactions, while 10% are idiopathic. The disease is classified according to body surface area (BSA) involvement and the thickness of epidermal loss. We report a case of a female with borderline personality disorder on antipsychotic medication, who developed SJS/TEN overlap after taking ciprofloxacin for her urinary tract infection (UTI). Her condition improved with meticulous management, but after switching her antibiotic from intravenous clarithromycin to oral linezolid, she developed SJS/TEN again, this time with more severe involvement. She received active management involving a multidisciplinary approach. Her condition improved slowly and, after one month, her lesions began to heal, and she was discharged with advice not to use both antimicrobial drugs in the future.

## Introduction

SJS/TEN (Stevens-Johnson syndrome/toxic epidermolysis necrosis) is a rare, acute, lethal skin condition characterized by sheet-like sloughing of skin and mucosa. Stopping the causative substance, followed by supportive treatment and adequate wound management in a specialist burns unit, is crucial in the treatment of patients with both TEN and SJS [1]. Erythematous macules and hemorrhagic mucous membrane erosions are common manifestations [2]. These disorders can also cause epidermal detachments of variable severity. These disorders have a variety of etiologies, the most prevalent of which is a reaction to medications [3,4]. It often begins with an "influenza-like" prodromal phase (malaise, fever), followed by painful cutaneous and mucous membrane lesions (ocular, oral, and vaginal) and other systemic symptoms [1]. Infections, drug-induced, malignancy-related, and idiopathic are the four etiological groups. In adults and the elderly, drugs and cancer are frequently implicated as the etiology. Infections are commonly associated with pediatric cases [5].

This case report is unique in that the patient had a borderline personality disorder and experienced SJS/TEN twice due to the intake of distinct antimicrobial medication classes. Several cases of SJS/TEN due to ciprofloxacin have been reported in the literature [6-8]. However, only 63 linezolid-related SJS reports have been identified so far, affecting males and females equally [9]. The severity of illness score for toxic epidermal necrolysis (SCORTEN), initially developed in 2000, is used to determine the severity of the condition [10]. In a 1996 patient cohort, SCORTEN outperformed the ABCD-10 [age, bicarbonate levels, cancer, dialysis-body surface area (BSA>10%)] score in predicting prognosis in SJS/TEN [10].

## Case presentation

A 44-year-old unmarried female, who was a known case of diabetes mellitus type 1 for 30 years, controlled on insulin 70/30 (23 units in the morning and 20 units at night), and borderline personality disorder for three years, on topiramate 50 mg twice a day for one year along with antidepressant bupropion 3 mg at night for one year and melatonin 3 mg at night for six months, presented in the emergency with complaints of fever, sore throat, generalized redness, and painful peeling of skin from cheeks, lips, hand and feet, and oral erosions. She gave a history of burning micturition and fever four days ago, for which she had been prescribed the tablet ciprofloxacin 500 mg once daily; 18 hours after taking the second dose of ciprofloxacin 500 mg, she developed these lesions.

**Figure 1:**
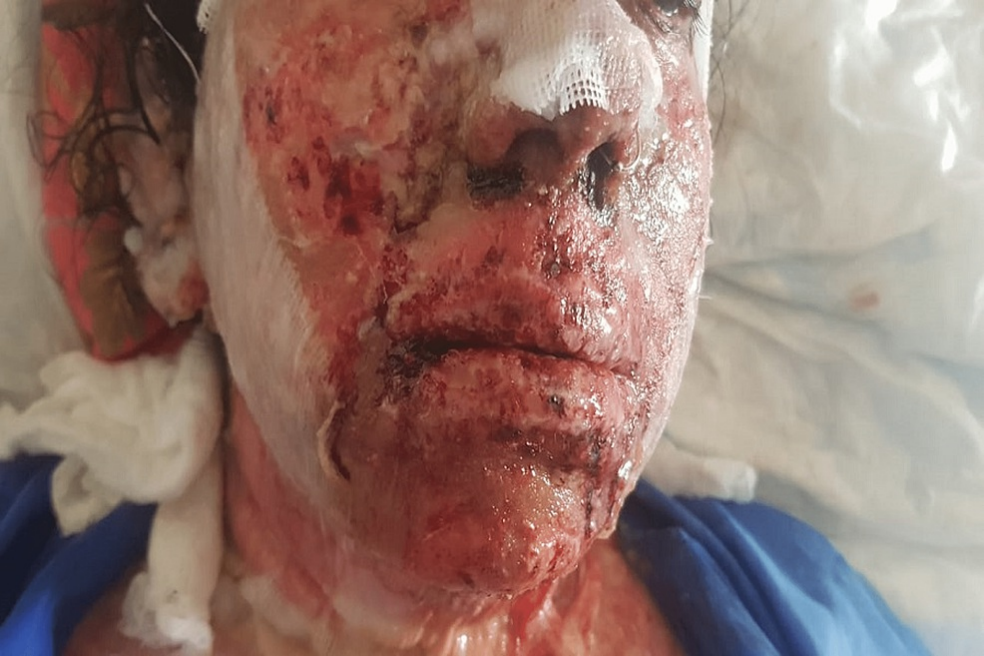
Erosion and swelling on face and lips, with hemorrhagic crusting on lips and nose

**Figure 2:**
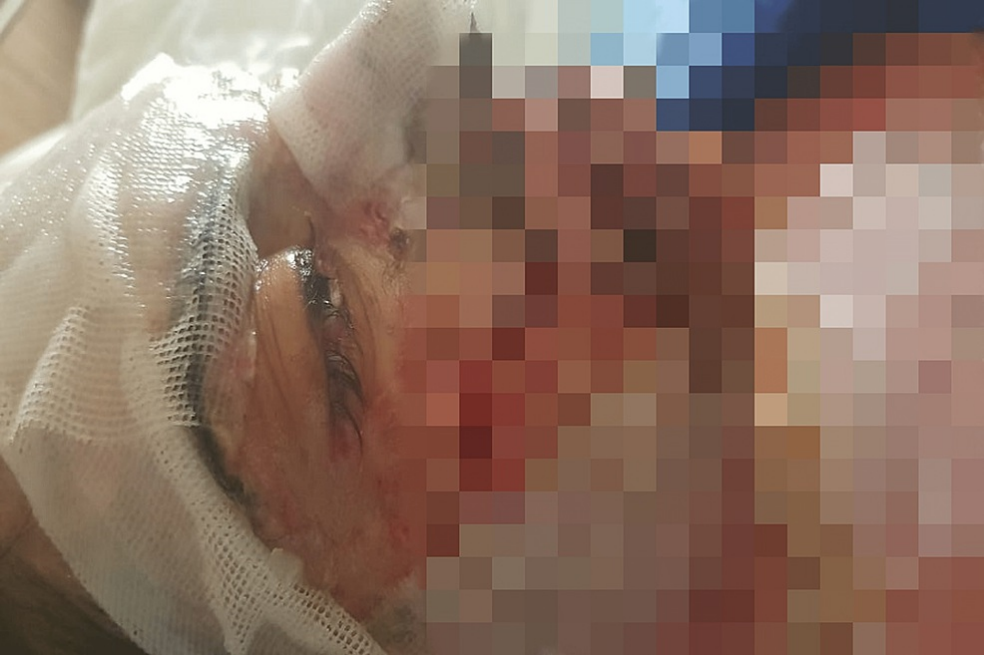
Visible swelling on eyes

On examination, there was erythema of hands, feet, and face. Her face and lips were swollen, and the next day, vesicles and tense bullae started to form, with loss of skin from hands, feet, and lips. Painful erosions were present on the lips, tongue, and soft palate with the formation of hemorrhagic crusts around the lips (Figures 1, 2). Purpuric macules on the abdomen with few atypical target-like lesions on the legs were noted. The total BSA involved was 11%. She had watery eyes with itching, but no redness was appreciated. Nikolsky's sign was positive. A diagnosis of SJS/TEN overlap was made. Ciprofloxacin 500 mg was stopped along with topiramate 50 mg, bupropion 3 mg, and melatonin 3 mg, which the patient had been taking for borderline personality disorder. Her vital signs showed a BP of 100/60 mmHg, a pulse of 102 beats/minute, a respiratory rate of 21 breaths/minute, and a temperature of 102 °F; her random blood sugar (RBS) level was 169 g/dl and oxygen saturation was 97%. Systemic examination was normal.

Investigations were done, and the patient's complete blood picture showed hemoglobin of 10.7 g/dl, normocytic and normochromic, and a platelet count of 121,000, but no eosinophilia was noted. However, the absolute neutrophil count was elevated. C-reactive protein (CRP) levels were elevated at 8.9 mg/dL. Prothrombin time (PT) and activated partial prothrombin time (aPTT) were within normal ranges. Liver function tests (LFTs) were deranged with normal bilirubin, raised alkaline transaminase of 231 IU/L, and alkaline phosphatase level of 156 IU/L. HbA1C was 6.89, and urea and creatinine were within the normal range. Electrolytes were deranged with sodium of 118 mmol/L (134-154 mmol/l) and potassium of 5.37 mmol/L (3.4-5.0 mmol/l), and bicarbonate levels were 14.5 mmol/L (23-27 mmol/l). HBsAg and anti-HCV came out to be negative. Chest X-ray, electrocardiogram (ECG), two-dimensional echocardiogram (2D Echo), and ultrasound abdomen were normal. A routine urine examination showed few leucocytes in urine. Blood culture and urine cultures did not grow any organism. Serum total protein was decreased along with serum albumin. SCORTEN was calculated to be 4 with a mortality rate of 58%. The patient refused a skin biopsy.

ICU, emergency medicine, and psychiatry departments were taken on board. The patient was started on the following management plan as per a multidisciplinary approach: injection methylprednisone 500 mg in 500 ml normal saline to be administered every six hours for three days, injection clarithromycin 500 mg in 500 ml normal saline IV twice a day, injection human albumin 20% 100 ml one time daily for three days, injection hydroxyzine 25 mg intramuscular thrice a day, polymyxin B skin ointment to be applied on erosions along with a daily dressing of paraffin-embedded gauze, and emulsifying ointment on the whole body twice daily. After consulting an ophthalmologist, a tear substitute, methylprednisolone eye drops, was applied, with two drops in each eye two times/day for three days. The patient was advised to apply miconazole oral gel B.D. on erosions along with nystatin drops and 0.01% potassium permanganate mouthwash for oral care. Nutritionists advised a high-caloric diet with high protein, low fat, and carbohydrates. A type C blenderized diet was started. Blood sugar monitoring was done for four hours, and blood sugar levels were controlled with an insulin injection according to a sliding scale. Input and output monitoring was done daily. A gynecology consult was also done to rule out genital involvement.

**Figure 3:**
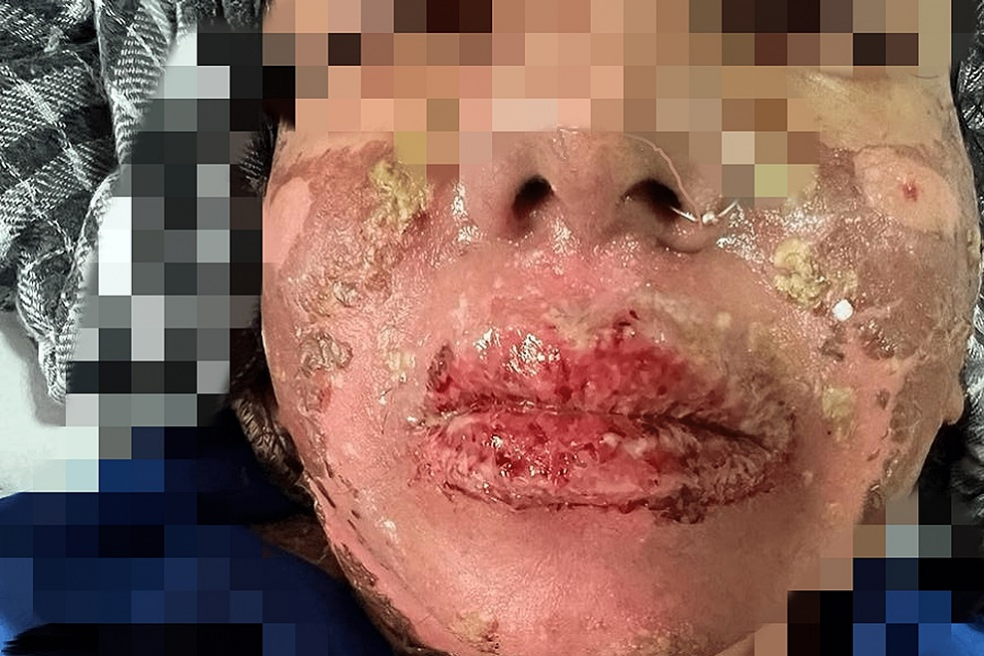
Decreased swelling on face and lips with the healing of erosions

**Figure 4:**
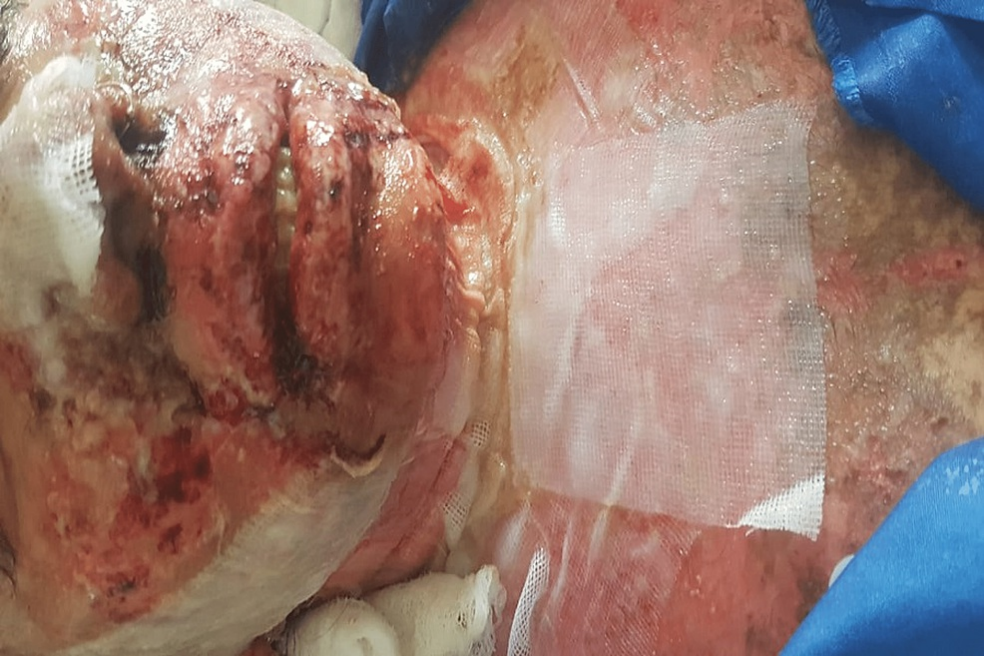
Worsening of swelling on face, neck, and chest after taking the first dose of linezolid 600 mg

The patient's physical and lab parameters started to improve on the third day of admission (Figure 3); she started consuming orally and was started on oral prednisolone 5 mg two tablets in the morning and in the afternoon. The antibiotic was switched from intravenous clarithromycin 500 mg to oral linezolid 600 mg twice daily. The patient then became agitated, unable to sleep, and was showing bizarre behavior. Her condition deteriorated, and oral erosions worsened four hours after taking a single dose of tab linezolid 600 mg. She was unable to open her mouth properly. Hands and feet became swollen, and an IV line could not be maintained. Blisters started to develop on the neck with the sloughing of skin (Figure 4). Eye redness and swelling were observed, as well as the development of extreme genital erosions along with pus discharge. BSA involved was 14%.

She was shifted to the burn ICU, and a femoral central venous catheter (CVP) was placed. Her vital signs deteriorated: BP dropped to 100/60 mmHg; her pulse increased to 109 beats/minute, her respiratory rate was 22 breaths/minute, her oxygen saturation decreased to 92%, and her RBS was found to be 190 gm/dl. Lab parameters also became deranged. CBC showed increased absolute neutrophil count, and CRP level was raised at 10.5 mg/dL; urea and creatinine levels were normal, serum sodium was 124 mmol/l, potassium was 4.2 mmol/l, and bicarbonate was 16 mmol/l. PT and aPTT were normal.

**Figure 5:**
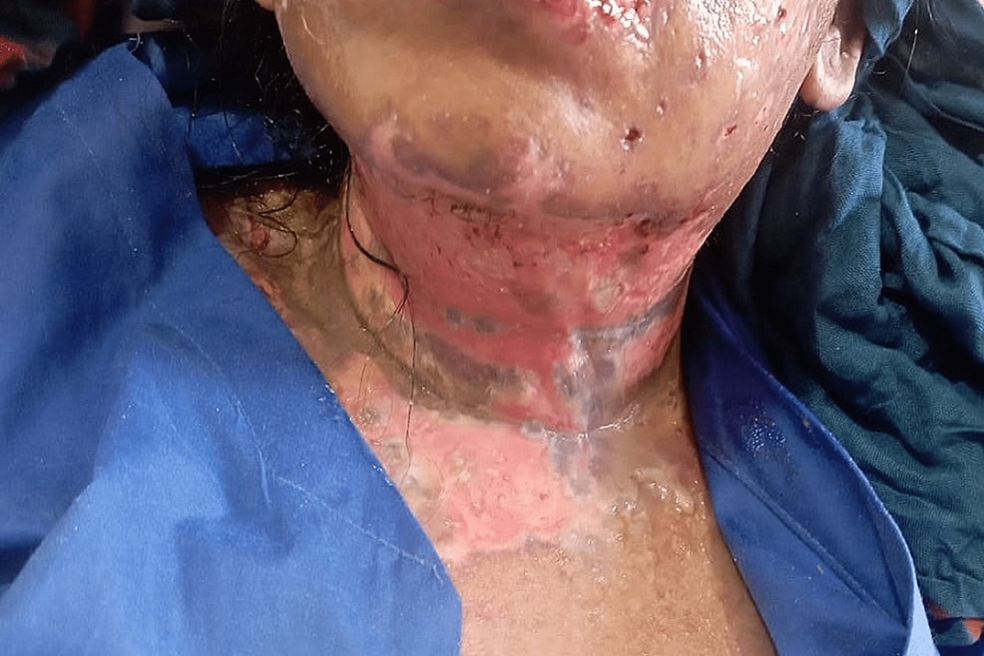
Improvement in the swelling of neck and chest, with healed erosions on the day of discharge

**Figure 6:**
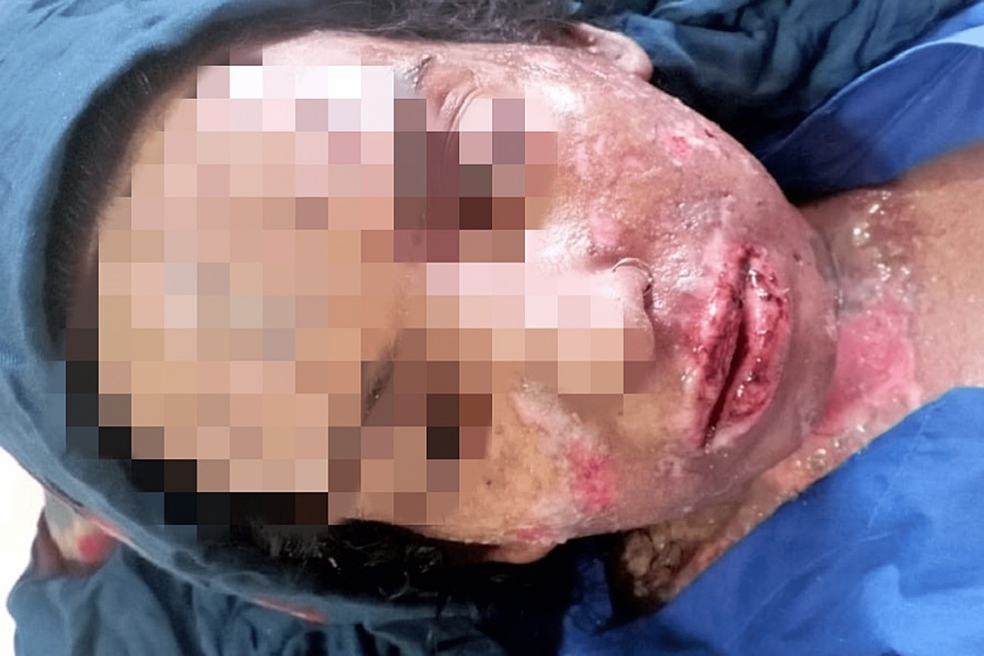
Healed erosion of lips, cheeks, and neck on the day of discharge

SCORTEN calculated was 4 with a mortality rate of 58%, and the ABCD-10 score was 2 with a mortality rate of 12.3%. The patient was started on IV hydrocortisone 200 mg three times a day, and linezolid was stopped. Multidisciplinary care was given by involving medical, ENT, eye, psychiatry, gynecology, and dermatology departments, with meticulous skin care as well as oral and genital lesion care. She started to show improvement gradually after the second week of the hospital stay. It was tough to treat her because of her borderline personality disorder. Steroids were tapered off slowly from the third week onwards. She was discharged after one month when her lesions healed (Figures 5, 6) and her laboratory parameters became normal. Her discharge plan mentioned that she should never take fluoroquinolones and linezolid in the future.

## Discussion

Adverse drug reactions (ADRs) are one of the primary causes of mortality in hospitalized patients, accounting for 0.3-7% of all hospitalizations. These can range from minor rashes to serious reactions like SJS [11]. Erythema multiforme (EM), SJS, SJS/TEN overlap, and TEN are all part of the SJS continuum, referred to as delayed hypersensitivity syndrome. Patients are classified into three categories depending on the degree of skin detachment [3], but the international classification is based on the affected BSA; SJS affects less than 10% of BSA, SJS/TEN overlap involves between 10 and 30%, and TEN affects more than 30% of the BSA [1].

Medications are the most prevalent cause of TEN/SJS. They generally cause illness in children and adults within eight weeks; however, the median exposure duration is four days to four weeks. A history of tolerated pharmaceutical usage makes it less likely to be a trigger, as in our case where the patient was taking selective serotonin reuptake inhibitors (SSRIs) and melatonin. Non-steroidal anti-inflammatory drugs (NSAIDs), allopurinol, anticonvulsants such as lamotrigine, phenytoin, and carbamazepine, antimicrobial sulfonamides, and the antiviral nevirapine are other common pharmaceutical triggers [1,12]. Antibiotics such as amoxicillin, ciprofloxacin, and doxycycline are less strongly related, and there is a possible relationship with other medicines such as pantoprazole, glucocorticoids, and terbinafine to mention a few [13]. Targeted immunotherapy and more traditional cancer medicines, such as vemurafenib, ipilimumab, pembrolizumab, nivolumab, thalidomide, and tamoxifen, have also been linked to TEN/SJS [14]. Infection with Mycoplasma pneumonia is the second most prevalent cause of SJS, especially in children. However, a trigger has not been observed in more than one-third of the instances [15]. Other triggers, such as herbal medicine vaccines, systemic disorders, and contrast agents, have been documented; however, a strong relationship with these agents has not been found [15].

Epidermal necrosis is caused by two types of mechanisms: extrinsic and intrinsic. Cytotoxic lymphocytes, monocytes, granulysin, perforin, granzyme, and Fas/Fas ligand interactions are part of the extrinsic route. Intrinsic refers to keratinocytes producing hazardous compounds, which subsequently create reactive oxygen species and eventually lead to the generation of tumor necrosis factor-alpha, inflicting more harm [5,16,17]. To determine the severity of the condition, many scores are utilized, including SCORTEN, initially published in 2000, which incorporates parameters such as age (>40 years), malignancy, tachycardia (>120 bpm), blood glucose (>14 mM), and serum bicarbonate (10 mM). The SCORTEN predicts mortality quite well. Other scores, including the modified APACHE II (acute physiology and chronic health evaluation-II) and ICNARC (Intensive Care National Audit and Research Center) ratings, and ABCD-10, have been shown to be inferior to SCORTEN in predicting mortality in SJS/TEN [10].

A prodrome of malaise, sore throat, and fever for many days precedes a quickly developing macular exanthem with purpuric centers that consolidate along with mucosal involvement in TEN/SJS. Atypical targets might also be found. Following this, the skin becomes highly painful, blistering, and peels the epidermis with little pressure (known as Nikolsky's sign) [18]. At least two mucosal surfaces are affected, including the eyes (conjunctivitis, less frequently corneal ulceration, anterior uveitis, panophthalmitis), the lips/mouth (cheilitis, stomatitis, painful mouth ulcers), the pharynx and esophagus (causing difficulty eating), the genital area and urinary tract (erosions, ulcers, urinary retention), the upper respiratory tract (trachea), and gastrointestinal system (diarrhea). Dehydration and severe malnutrition are other complications, as are shock and multiple organ failure, including renal failure, thromboembolism, sepsis, and disseminated intravascular coagulation. Blindness, cataract, and stricture development in oral and vaginal erosions are examples of chronic consequences.

Treatment

The treatment of the condition involves the following approaches:

First Line

(1) Withdraw the culprit drug; (2) if the epidermal loss is >10% BSA, transfer to a specialist unit (ICU or burns unit); (3) institute supportive care package, with particular attention to a heated environment, fluid replacement, nutritional regimen, analgesia, and preventing and treating infection; (4) specialist skin care nursing is essential for the delivery of topical therapy and dressings.

Second Line

In the early stages of the acute phase, consider using IVIG (0.5-1 g/kg daily for three to four consecutive days) or systemic corticosteroids (e.g., prednisolone 0.5-1 mg/kg daily for 10 days, and then tapered; or IV methylprednisolone 500 mg on three consecutive days), or ciclosporin (3 or 4 mg/kg/day in divided doses for 10 days, and then tapered) [19].

Our patient developed the first episode of SJS/TEN overlap after taking ciprofloxacin. We hypothesized that as the patient was using the tablet topiramate 50 mg for borderline personality disorder, it got metabolized by CYP3A4 (cytochrome 3A4) in the liver, which was potentially inhibited by ciprofloxacin [8], thereby causing an increase in the levels of antipsychotic topiramate. The combined effect of these two drugs increased the chances of the patient developing SJS/TEN. BSA involved was 11% after the first attack. After proper ICU and medical care, the patient recovered. Her skin and oral lesions started to settle. When her antibiotic was switched from intravenous clarithromycin to oral linezolid, she had the second episode of SJS/TEN overlap after starting linezolid 600 mg, which is reported to cause SJS/TEN less frequently [20]. BSA affected this time was 14%, with involvement of oral, eye, and genital mucosa and extensive neck and chest involvement. She was managed with gentle care in the burn ICU along with a multidisciplinary approach and her condition gradually improved in two weeks. Her lesions started to heal after the third week, and she was discharged from the hospital after one month. She was followed up for three months to look for any chronic complications of the disease; her lesions healed, leaving behind post-inflammatory pigmentation,` which also started to settle after the fourth month.

## Conclusions

SJS/TEN is a severe life-threatening condition with skin, mucosa, and multisystem involvement. Early diagnosis and treatment are critical for reducing mortality. Our patient already had multiple comorbidities, such as diabetes mellitus type 1 and borderline personality disorder, and first developed SJS/TEN after taking ciprofloxacin. She started to recover after stopping all known culprit drugs and receiving extensive ICU management but again developed SJS/TEN with more severe skin and mucosal involvement as a reaction to a relatively safer antibiotic, linezolid. This case study shows that great caution should be taken while prescribing ciprofloxacin and then switching to linezolid in patients and ADRs should always be kept in mind. Hence, giving a test dose of these antimicrobial agents should always be considered in every patient before administering them regularly.
